# Milk–cereal mix supplementation during infancy and impact on neurodevelopmental outcomes at 12 and 24 months of age: a randomised controlled trial in India

**DOI:** 10.1017/S0007114522003944

**Published:** 2023-09-14

**Authors:** Ravi Prakash Upadhyay, Sunita Taneja, Tor Arne Strand, Mari Hysing, Beena Koshy, Nita Bhandari, Rajiv Bahl

**Affiliations:** 1 Centre for Health Research and Development, Society for Applied Studies, New Delhi, India; 2 Department of Global Public Health and Primary Care, Centre for International Health, University of Bergen, Bergen, Norway; 3 Department of Research, Innlandet Hospital Trust, Lillehammer, Norway; 4 Department of Psychosocial Science, Faculty of Psychology, University of Bergen, Bergen, Norway; 5 Department of Developmental Pediatrics, Christian Medical College, Vellore, India; 6 Department of Maternal, Newborn, Child and Adolescent Health, World Health Organization, Geneva, Switzerland

**Keywords:** Neurodevelopment, Infancy, Milk protein, Nutritional supplementation, Randomised controlled trial, India

## Abstract

Inadequate protein intake and lack of micronutrients may affect neurodevelopment in infants. This randomised controlled trial was conducted to measure the effect of two milk–cereal mixes with modest and high amounts of protein and enriched with multiple micronutrients, given between 6 and 12 months, on cognitive, language, motor and behavioural scores at 12 and 24 months of age, compared with no-supplementation. The two supplements were also compared with each other. The study was conducted in urban Delhi, India, and the infants were randomised in a 1:1:1 ratio to the three study groups. At 12 and 24 months of age, 1134 and 1214 children were available, respectively. At 12 months of age, compared with no-supplement group, an increase in the motor scores (mean difference, MD 1·52, 95 % CI: 0·28, 2·75) and a decrease in the infant temperament scores (MD − 2·76, 95 % CI: −4·23, −1·29) in the modest-protein group was observed. Those in the high-protein group had lower socio-emotional scores (MD − 1·40, 95 % CI: −2·43, −0·37) and higher scores on Infant Temperament Scale (MD 2·05, 95 % CI: 0·62, 3·48) when compared with modest-protein group. At 24 months, no significant differences in any of the neurodevelopment scores between the three study groups was found. In conclusion, supplementation with modest amount of protein and multiple micronutrients may lead to short-term small improvements in motor function and infant temperament. There appears no advantage of supplementing with high protein, rather negative effects on infant behaviour were observed

About 250 million under-five children in low- and middle-income countries (LMIC) do not reach their full developmental potential^(1)^. Sub-Saharan Africa (43·8 %) followed by South Asia (37·7 %) are the leading contributors^(1)^. The brain growth occurs maximally in the first 2–3 years of postnatal life, particularly during infancy^(2,3)^. Adequate nutrition plays an important role in promoting healthy brain growth and development^(4,5)^. Complementary feeding is usually inadequate in low-resource populations in low- and middle-income countries. The concerns are both with the quantity and quality of complementary foods, and the infants often fail to achieve an adequate intake of key nutrients for optimal growth and development^(6–9)^. A review on the quality of complementary foods in low-resource settings documented that about 50–75 % of the total protein a child eats is from cereals and other plant sources^(10)^. Evidence suggests that in diets deriving over 50 % of protein from cereal sources, protein quality is relatively poor, thereby limiting protein utilisation, which in turn may adversely impact overall growth and development^(9–11)^.

Proteins are specially required for brain development. They have a useful role to play in neurogenesis, neuronal migration and differentiation, synaptogenesis, oligodendrocyte myelination, neurotransmitter production and reuptake, and maintaining electrical efficiency^(5,12,13)^. Proteins obtained from dairy sources have been documented to increase the levels of insulin-like growth factor-1 which is a neurotrophic polypeptide playing a crucial role in growth, development and maturation of the central nervous system^(14–16)^. Supplementary Fig. 1 provides a conceptual framework through which supplementation with protein may promote neurodevelopment in children. Limited studies suggest an association of protein intakes in children with improvement in their neurodevelopment outcomes^(17–19)^. We currently do not fully understand the effects of supplementing with food that has higher amounts and improved quality of protein, that is, animal/milk source protein, to infants on their neurodevelopment. An effort to elucidate the usefulness of optimised nutritional interventions during the second half of infancy, that coincides with the period of complementary feeding, is required to guide the design of nutritional programmes for infants in low-middle-income settings. The present study aimed to test the effect of micronutrient-enriched milk–cereal-based supplements, differing in their protein content, provided to infants aged 6 months of age, for a period of 180 d, on neurodevelopmental outcomes at 12 months of age, when compared with no-supplementation. The intent was also to compare the two supplements with each other to understand whether increasing the amount and quality of proteins in the supplements led to a difference in neurodevelopmental outcomes. These infants were followed up without any supplementation in the period between 12 and 24 months, and their neurodevelopment was assessed again at 24 months of age. This was done to explore whether a nutritional intervention of short duration in early infancy can impact neurodevelopment in early childhood. The study is a part of a primary trial that assessed the impact of such nutritional supplementation during infancy on linear growth at 12 months of age^(20)^. In the primary trial, small improvement in length-for-age z scores (mean difference, MD 0·08), weight-for-age *z* scores (MD 0·12), weight-for-length *z* scores (MD 0·11) and mid-upper arm circumference *z* scores (MD 0·10) in the high-protein group was observed, when compared with no-supplement group.

## Materials and methods

### Study setting, design and participants

Details of the parent study have been published previously (CTRI/2018/04/012932)^(20)^. Infants enrolled in this parent trial were separately consented at 12 months for their neurodevelopmental assessments at 12 and 24 months and anthropometric assessments at 15, 18 and 24 months of age. The parent study was an individually randomised controlled efficacy trial conducted in low-resource settings in urban Delhi, India. Study subjects were infants aged 6 months (+29 d) who were breastfed. Infants not breastfed at the time of enrolment, those with documented illness requiring prolonged institutional management, with severe acute malnutrition (weight-for-height < –3 sd), with major congenital malformations and mother–infant dyads that were likely to move out of the study site within 6 months were excluded^(20)^.

### Screening and enrolment

A door-to-door survey was conducted to identify infants aged 6 months (+29 d). Those aged under 6 months were followed up periodically until they reached 6 months. The screening and enrolment team visited the family and explained the study to the mother and family members. If the infant was found eligible, consent for screening was obtained from the mother.

### Randomisation, allocation and blinding

Eligible infants were randomised to either one of the two intervention groups or the control groups (allocation ratio of 1:1:1) through a web-based system^(20)^. A randomisation list with blocks of variable length (i.e. 3 and 6) was used. Complete blinding of the study intervention delivery team and participants was not possible due to the nature of the intervention, that is, no milk–cereal mix supplemented in the control, but supplements provided to infants in two intervention groups. However, blinding was ensured for the two intervention groups that differed in the amount and quality of milk protein supplemented^(20)^. Two different sets, each having thirteen unique English language alphabets, were allotted to the two infant cereal mixes. An offsite person (Statistician from WHO, Geneva, Switzerland) not associated directly with the trial prepared the list of alphabets and their scheme of allocation. The two milk–cereal mixes were identical in packaging, taste, consistency and colour. The outcome assessment team comprising of psychologists were blinded to the group allocation.

### Study interventions

The details of the interventions, the nutritional composition of the milk–cereal mixes and the implementation strategy have been previously published in detail^(20)^. Infants in the control group received no milk–cereal mixes. Counselling was provided to mothers and family members by trained nutritionists on continued breast-feeding, optimal complementary feeding practices, infant care practices such as immunisation, early recognition and timely care-seeking for illness in all the three study groups. Infants were provided iron folic acid syrup (10 mg elemental iron and 100 mcg folic acid) in the three groups^(21)^. Infants in modest and high-protein groups received daily supplementation, for 180 d, with milk–cereal mix that provided about 125 kcal of energy, 30–45 % energy from fats and 80–100 % RDA of growth-relevant multiple micronutrients^(20)^. The difference in the supplement in these two groups was in the total amount of protein (modest group: 2·5 g protein, protein energy ratio of 8 %; high group: 5·6 g protein, protein energy ratio of 18 %) and absolute amount of protein from milk source (modest group: 30 % of the total protein from milk, i.e. 0·75 g; high group: 30 % of the total protein from milk, i.e. 1·68 g). The infant milk–cereal mix was designed in a way that it should provide about 50–60 % of the non-breast milk energy requirement. The cereal mixes were replenished on a weekly basis.

The intervention delivery team visited households to provide weekly supplies of milk–cereal sachets to mothers in both the intervention groups. During the weekly visits, this team gathered information on compliance by collecting packets of the mix and reinforced intake. The team collected information on the number of empty sachets and number of sachets with some of the mix remaining. As part of the study processes, the team collected information in their diaries on the consumed amount in the collected sachets, i.e. completely consumed, not at all consumed, half to three-fourth or less than half of the content consumed. This information was used to identify subjects with low compliance, and they were then visited by the team supervisor to discuss barriers to optimal intake. Some measures taken to prevent intra-household sharing were that the milk–cereal mix was promoted for use for young children and not for older children or adults. Further, in order to prevent sharing, biscuits/cookies were provided for other children in the household.

### Sample size and selection of participants

We considered a 0·25 sd MD (3·75 points, 1 sd = 15 points)^(22)^ in cognitive, motor and language scores at 12 months between the modest-protein group and the no-supplement group and a 0·30 sd (4·5 points) difference between the high-protein group and the no-supplement group. With 90 % power, two-sided 5 % *α* level and 20 % attrition, 400 infants per group were required for the comparisons between the modest-protein and no-supplement group and 280 infants per group for comparisons between high-protein group and no-supplement group. We, therefore, aimed to include a total of about 1200 infants for the assessment of neurodevelopment outcomes. With a sample size of 400 infants each in modest-protein and high-protein groups, we were powered at 80 % to detect a difference of 2·5 points (0·17 sd)^(22)^ in cognitive, motor and language scores between the two supplement groups. The 1200 infants were planned to be followed up for their neurodevelopment assessments at 24 months of age.

The children for neurodevelopment assessments were contacted in a consecutive manner, that is, as and when they completed their anthropometric and biochemical assessments in the primary trial at 12 months of age. The family members of these children were approached for their consent for participation in the neurodevelopment assessments at 12 and 24 months of age. For infants who could not be contacted at 12 months of age because the family members had temporarily moved out of the study area at the time of house visit or in those who had crossed the window period of +4 weeks at the time they were approached for inclusion in this study, the study team visited the house at the time of anthropometric assessments at 15 months of age and obtained written informed consent for neurodevelopment assessments at 24 months of age. Such children did not have their 12 months neurodevelopment assessments but were eligible for assessments at 24 months.

### Outcomes and their ascertainment

The primary outcomes were cognitive, motor and language scores at 12 and 24 months of age. The secondary outcomes were socio-emotional scores at 12 and 24 months of age, infant temperament scores at 12 months of age, and mean internalising and externalising behaviour scores at 24 months of age. A window period of +4 weeks was considered for the assessments to be conducted. Details of the data collection have been presented previously^(20)^. A 24-h dietary recall at 12 and 24 months of age was done in a subsample of infants and children undergoing neurodevelopmental assessments by trained nutritionists. Data on morbidities were collected for the previous 2 weeks for visits done at 9, 12 and 24 months of age. Anthropometric assessments were conducted by trained and standardised workers at 15, 18 and 24 months of age.

For the neurodevelopmental outcomes, an independent team of trained and standardised psychologists conducted the assessments. This team was blinded to the group allocation. For the primary outcomes, Bayley Scales of Infant and Toddler Development (BSID), 3rd edition was used^(22,23)^. This is a comprehensive assessment tool of developmental functioning in infants and toddlers aged 1–42 months. The process of adaptation of BSID for use in the study setting has been previously described^(24)^. The inter-rater agreement for the standardisation exercises before the start of the study as well as during the conduct of the assessments was excellent (intraclass correlation: 0·94–0·99). Infant temperament was assessed using Infant Temperament Scale, which is a parent-reported measure containing forty-seven items that assess six dimensions (activity, positive emotionality, negative emotionality, sociability, attention and soothability). Higher scores on Infant Temperament Scale reflect difficult temperament^(25)^. The scale has been adapted for use in low-middle-income setting and has been used previously in one of our recent studies^(24,25)^.

Behavioural problems were assessed using Child Behavior Checklist – preschool (CBCL). This is a caregiver-reported tool intended for children aged 18 months to 5 years^(26)^. It consists of 100 items, where the responses are recorded on a Likert scale. The responses are summed to provide a score for internalising and externalising behavioural problems. A total score from all questions is derived by adding up the internalising scores, externalising scores, scores pertaining to sleep problems and other problems. The raw scores are converted into t-scores, and increasing t-scores indicate the behavioural problems in a child. The tool has been used in previous research conducted in similar settings^(24,25,27,28)^.

We also measured home environment and child stimulation by caregivers. Home environment at 12 months of child age was assessed using ‘Pediatric Review of Children’s Environmental Support and Stimulation (PROCESS)’ questionnaire^(29,30)^. It consists of three components: a parent questionnaire, clinical observation and a toy checklist. Total scores are summed across the three sections, and higher scores reflect better stimulation and support to infants. For assessing home environment and stimulation at 24 months of age, we used the Home Observation for Measurement of the Environment (HOME) tool for infants and toddlers^(31)^. Both the PROCESS and HOME tools were used after adapting according to local cultural context, translating in local language (Hindi) and pre-testing for use.

### Statistical analysis

All analyses were done using STATA version 16.0 (Stata-Corp LLC). We calculated the means (sd) or median (IQR, interquartile range) for continuous variables and proportions for categorical variables. Means (standard error, se) of dietary intakes for energy, carbohydrates, protein and fats for children in each of the three groups were calculated using data from a single 24-h dietary recall at 12 and 24 months of age.

The primary analysis included the comparison of neurodevelopment outcomes at 12 and 24 months of age between the three study groups and was based on the intention-to-treat principle. Effect sizes (difference in means and 95 % CI) for the continuous outcomes were calculated using generalised linear models of the Gaussian family with an identity-link function. The primary analysis was unadjusted as there were no significant differences in the baseline characteristics among children in the three groups. Additionally, we also conducted an adjusted analysis after including variables in the models that have been shown to influence neurodevelopment outcomes, based on previously published studies^(1,32–34)^. We used generalised estimating equation models when the outcomes were measured more than once for the children. We used generalised estimating equation models of the Gaussian family with an identity-link function, an autoregressive covariance–variance matrix that factored in time and calculated robust standard errors. Although not an *a priori* decision, we conducted subgroup analysis with infants who were stunted (length-for-age z-scores, LAZ < –2) at the time of enrolment in the study in order to explore whether there were any differential effects of supplementation on neurodevelopment outcomes at 12 and 24 months of age in these high-risk infants. For this subgroup analysis, we adjusted for variables that were selected based on prior literature and biological plausibility to influence child development outcomes. This adjustment was done as the original randomisation was not preserved when utilising this subgroup.

## Results

**Fig. 1.**
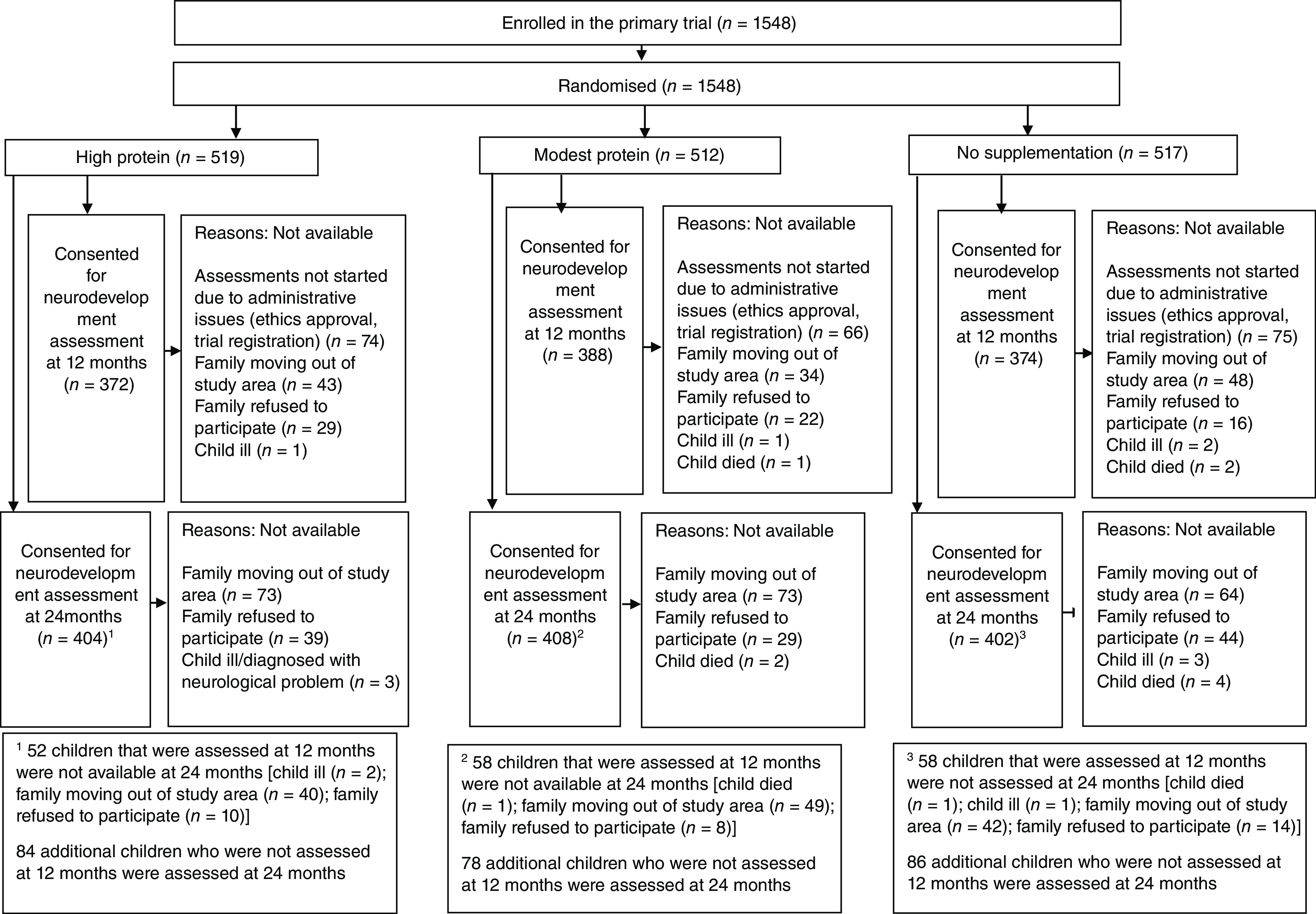
Trial profile. For 12 months of neurodevelopment assessment, some infants had crossed the window period of +4 weeks at the time they were approached for consenting. For these children, the study team visited the house at the time of anthropometric assessments at 15 months of age and obtained written informed consent for neurodevelopment assessments at 24 months of age.

The primary trial assessed the impact of milk–cereal mix supplementation on linear growth at 12 months in a total of 1548 infants^(20)^. From these, 1134 infants were assessed for their neurodevelopment at 12 months (high protein, *n* 372; modest protein, *n* 388; no supplement, *n* 374) and 1214 children (high protein, *n* 404; modest protein, *n* 408; no supplement, *n* 402) at 24 months of age (Fig. 1). The reasons for non-participation were mainly related to families moving out of the study area, refusing to participate, or that the child had crossed the window period of +4 weeks at the time of contact (Fig. 1). Findings on compliance to the milk–cereal mix and iron folic acid (IFA) among the three groups have been presented previously^(20)^. The proportion of infants consuming milk–cereal mix on *>* 75 % of days of the 180 d supplementation period was > 80 % for both modest-protein and high-protein group. The proportion of infants who consumed iron folic acid for *>* 75 % of days was about 70 % for the three groups. The baseline characteristics (i.e. at the time of enrolment in the primary trial) of the children assessed for their neurodevelopment at 12 and 24 months of age were statistically similar across the three groups (Table 1). The mean (sd) LAZ and weight-for-age z scores (WAZ) at 24 months of age were statistically similar among the three groups (LAZ: –1·36 (0·99), –1·41 (1·01), –1·43 (1·05) and WAZ: –1·37 (0·98), –1·40 (1·07), –1·42 (0·99) for the high-protein, moderate-protein and no-supplement group, respectively). The mean (sd) PROCESS score at 12 months (125·0 (11·9); 125·5 (12·7); 125·0 (12·8)) and HOME score at 24 months (39·1 (5·5); 38·9 (4·9); 39·0 (5·3)) were statistically similar among the high-protein, modest-protein and no-supplement group, respectively.

**Table 1.** Baseline characteristics of the children assessed and their families, by the study groups

	Children assessed at 12 months						Children assessed at 24 months					
	Modest-protein group (*n* 388)		High-protein group (*n* 372)		No-supplement group (*n* 374)		Modest-protein group (*n* 408)		High-protein group (*n* 404)		No-supplement group (*n* 402)	
	*n*	%	*n*	%	*n*	%	*n*	%	*n*	%	*n*	%
Infant characteristics												
Proportion of male infants	205	52·8	178	47·9	198	52·9	200	49·0	189	46·8	209	52·0
LAZ scores*												
Mean	–1·21		–1·15		–1·20		–1·19		–1·12		–1·21	
SD	1·12		1·92		1·02		1·0		1·04		0·99	
Stunted (< –2 LAZ)	89	22·9	76	20·4	78	20·9	92	22·6	81	20·1	82	20·4
WLZ scores*												
Mean	–0·44		–0·44		–0·48		–0·43		–0·37		–0·49	
SD	1·02		1·06		1·03		1·02		1·09		1·01	
Wasted (< –2 WLZ)	23	5·9	33	8·9	24	6·4	23	5·6	29	7·2	21	5·2
WAZ scores*												
Mean	–1·13		–1·10		–1·15		–1·12		–1·02		–1·17	
SD	1·05		1·14		1·09		1·05		1·08		1·03	
Underweight (< –2 WAZ)	75	19·3	68	18·3	81	21·7	80	19·6	66	16·3	88	21·9
Birth order												
Mean	2·18		2.20		2·15		2·14		2·16		2·15	
SD	1·13		1·32		1·31		1·13		1·22		1·22	
Socio-demographic characteristics												
Wealth quintile												
Poorest	70	18·0	69	18·6	62	16·6	73	17·9	70	17·3	74	18·4
Very poor	77	19·9	68	18·3	83	22·2	75	18·4	72	17·8	88	21·9
Poor	87	22·4	72	19·3	75	20·1	95	23·3	77	19·1	80	19·9
Less poor	67	17·3	79	21·2	82	21·9	76	18·6	95	23·5	90	22·4
Least poor	87	22·4	84	22·6	72	19·2	89	21·8	90	22·3	70	17·4
Maternal characteristics												
Age (years)												
Mean	25·2		25·4		25·3		24·9		25·4		25·2	
SD	4·1		3·9		4·2		3·9		4·0		4·1	
Duration of schooling (in years)												
Median	8		8		8		8		8		8	
IQR	5–10		3·5–10		4–10		4·5–10		4·5–10		3–10	
Never been to school	75	19·3	81	21·8	74	19·8	81	19·9	88	21·8	90	22·4
Homemakers	364	93·8	350	94·1	352	94·1	383	93·9	379	93·8	379	94·3
Paternal characteristics												
Age (in years)												
Mean	28·8		29·0		28·9		28·5		29·1		28·8	
SD	4·8		4·5		4·8		4·6		4·7		4·6	
Duration of schooling (in years)												
Median	8		8		9		8		8		8·5	
IQR	5–10		5–10		6–12		5–10		5–10		5–10	
Unemployed	5	1·3	11	3·0	8	2·1	6	1·5	11	2·7	8	2·0

LAZ, length-for-age z scores; WLZ, weight-for-length z-scores; WAZ, weight-for-age z-scores; IQR, interquartile range.

Values are mean and standard deviation unless reported otherwise.

* Calculated using WHO standards.

The findings of the analysis for the effect of supplementation on neurodevelopment outcomes at 12 months of age have been presented in Table 2. Compared with no-supplement group, there was an increase in the motor scores in the modest-protein group (MD 1·52, 95 % CI: 0·28, 2·75) but not in high-protein group (Table 2). No difference in motor scores was found for comparison between modest- and high-protein groups. There were no significant differences in the cognitive and language scores for any of the three comparisons, i.e. modest-protein *v*. no-supplement group, high-protein *v*. no-supplement group and modest-protein *v*. high-protein group. Those in high-protein group had lower socio-emotional scores when compared with modest-protein group (MD − 1·40, 95 % CI: −2·43, −0·37) (Table 2). Analysis of Infant Temperament Scale scores revealed lower scores for modest-protein group compared with no-supplement group (MD − 2·76, 95 % CI: −4·23, −1·29) and higher scores for high-protein group compared with modest-protein group (MD 2·05, 95 % CI: 0·62, 3·48) (Table 2). Similar findings were observed in the adjusted analysis (online Supplementary Table 1)

**Table 2.** Effect of infant nutritional supplementation on neurodevelopment outcomes at 12 months of age

							Mean difference (95 % CI)					
	Modest-protein group (*n* 388)		High-protein group (*n* 372)		No-supplement group (*n* 374)		Modest-protein group *v*. no-supplement group (Ref)		High-protein group *v*. no-supplement group (Ref)		High-protein group *v*. modest-protein group (Ref)	
	Mean	SD	Mean	SD	Mean	SD	Mean difference	95 % CI	Mean difference	95 % CI	Mean difference	95 % CI
BSID composite scores												
Cognitive	105·3	10·0	105·1	10·0	104·2	11·3	1·09	–0·44, 2·61	0·91	–0·63, 2·45	–0·18	–1·60, 1·25
Motor	95·7	8·4	94·9	9·1	94·2	8·9	1·52	0·28, 2·75*	0·77	–0·53, 2·06	–0·75	–1·99, 0·49
Language	95·6	8·4	94·8	8·6	94·6	8·9	1·02	–0·22, 2·26	0·22	–1·04, 1·49	–0·80	–2·01, 0·42
Socio-emotional	98	7·5	96·6	7·0	97·1	7·9	0·88	–0·22, 1·97	–0·53	–1·60, 0·55	–1·40	–2·43, −0·37†
Infant temperament												
Total infant temperament scores	109·2	9·7	111·2	10·4	111·9	10·9	–2·76	–4·23, −1·29†	–0·71	–2·24, 0·82	2·05	0·62, 3·48†

BSID, Bayley Scales of Infant and Toddler Development; CI, confidence interval.

* Statistically significant at *P* < 0·05.

† Statistically significant at *P* < 0·01.

At 24 months of age, there were no significant differences in the cognitive, motor, language and socio-emotional scores as well as in the scores for internalising behaviour, externalising behaviour and total problem for any of the three comparisons (Table 3; online Supplementary Table 2). In the generalised estimating equation analysis, compared with no-supplement group, only children in the modest-protein group had some improvement in their motor scores over the 12 months follow-up period (i.e. from 12 to 24 months of age) (MD 0·98, 95 % CI: 0·06, 1·91) (Table 4). There were no significant differences in the changes in cognitive and language scores across the 12 months follow-up period for modest- and high-protein groups, when compared with no-supplement group (Table 4). No significant differences in cognitive, motor and language scores were found for generalised estimating equation-based comparisons between modest- and high-protein groups.

**Table 3.** Effect of infant nutritional supplementation on neurodevelopment outcomes at 24 months of age

							Mean difference (95 % CI)					
	Modest-protein group (*n* 408)		High-protein group (*n* 404)		No-supplement group (*n* 402)		Modest-protein group *v*. no-supplement group (Ref)		High-protein group *v*. no-supplement group (Ref)		High-protein group *v*. Modest-protein group (Ref)	
	Mean	SD	Mean	SD	Mean	SD	Mean difference	95 % CI	Mean difference	95 % CI	Mean difference	95 % CI
BSID composite scores												
Cognitive	92·1	7·4	92·1	6·8	91·7	6·9	0·38	–0·60, 1·36	0·38	–0·57, 1·32	0·00	–0·98, 0·97
Motor	94·4	8·1	94·3	8·3	94·0	8·3	0·35	–0·78, 1·48	0·34	–0·81, 1·49	–0·01	–1·14, 1·11
Language	88·9	9·8	89·8	9·6	89·8	9·5	–0·92	–2·25, 0·40	–0·08	–1·39, 1·24	0·84	–0·48, 2·18
Socio-emotional	104·9	17·1	105·6	16·2	105·1	16·9	–0·14	–2·49, 2·22	0·43	–1·86, 2·72	0·57	–1·73, 2·86
Child behaviour checklist scores												
Internalising behaviour t-scores	36·6	7·2	36·2	7·0	35·8	6·6	0·80	–0·15, 1·75	0·40	–0·54, 1·34	–0·40	–0·58, 1·38
Externalising behaviour t-scores	44·7	10·4	45·2	9·8	45·5	9·6	–0·82	–2·20, 0·56	–0·40	–1·75, 0·94	0·42	–0·97, 1·82
Total problem t-scores	39·6	8·5	39·4	7·5	39·3	7·4	0·24	–0·86, 1·34	0·03	–0·99, 1·06	–0·21	–1·31, 0·90

BSID, Bayley Scales of Infant and Toddler Development; CI, confidence interval.

**Table 4.** Effect of supplementation with milk–cereal mix during infancy on cognitive, motor and language scores between 12 and 24 months of age using a GEE model

BSID composite scores	Modest-protein group *v*. no-supplement group (Ref)		High-protein group *v*. no-supplement group (Ref)		High-protein group *v*. modest-protein group (Ref)	
	Mean difference	95 % CI	Mean difference	95 % CI	Mean difference	95 % CI
Cognitive	0·79	–0·23, 1·80	0·61	–0·40, 1·61	–0·18	–1·17, 0·82
Motor	0·98	0·06, 1·91*	0·63	–0·34, 1·59	–0·35	–1·29, 0·58
Language	0·10	–0·95, 1·15	0·12	–0·94, 1·17	0·01	–1·03, 1·04

GEE, generalised estimating equation; BSID, Bayley Scales of Infant and Toddler Development.

Values are mean differences with 95 % CI, adjusted for time.

* Statistically significant at *P* < 0·05.

The proportion of children with morbidities was similar across the three groups at 9, 12 and 24 months of age (online Supplementary Tables 3 and 4). Supplementary Table 5 presents the findings from the dietary recalls at 12 and 24 months of age from a small subsample of children. At 12 months, the total energy, fat and carbohydrate consumption by infants was statistically similar in the three groups. The amount of protein consumed significantly differed among the groups, with highest intake in the high-protein group. Overall, the total energy consumed was lower, whereas the total amount of protein consumed was higher than the RDA among infants in all the three groups. At 24 months, the total energy, fat and protein consumption by children was statistically similar in the three groups. The amount of carbohydrate consumed differed among the groups, with highest intake in the no-supplement group and lowest in high-protein group. Even at 24 months of age, the total energy consumed was lower and the total amount of protein consumed was higher than the RDA among infants in all the three groups.

The subgroup analysis among infants stunted at the time of enrolment in the trial suggested a significant beneficial effect in modest-protein group, compared with no-supplementation group, on cognitive, motor and language scores at 12 months of age (online Supplementary Table 6). Compared with infants in the modest-protein group, those in the high-protein group had significantly lower cognitive, language and socio-emotional scores at 12 months of age. No differences were observed in cognitive, motor, language and socio-emotional scores among the three groups at 24 months of age (online Supplementary Table 6).

## Discussion

The current study was conducted to measure the effect of supplementing infants, for a period of 180 d, with micronutrient-enriched milk–cereal mixes containing varying amounts of protein (modest and high) on their neurodevelopment at 12 and 24 months of age. We found that compared with no supplementation, those receiving modest amount of protein had better motor scores (about 0·17 sd, considering 1 sd of 8·9 points in no-supplementation group) and less difficult temperament at 12 months of age. At this time point, the cognitive, motor, language and socio-emotional scores were similar between the high-protein and no-supplement group. The socio-emotional scores were lower and infant temperament scores higher, reflecting difficult temperament, for infants in the high-protein group compared with the modest-protein group. At 24 months of age, there was no effect of the intervention on any of the outcomes.

Existing direct evidence on the effect of protein supplementation during infancy and early childhood on neurodevelopment is limited and makes it difficult to arrive at a consensus. In most of the available studies, there is a lack of clear specification of the source of animal protein being investigated (i.e. milk or meat based)^(35)^. Further, the age range of children being studied is diverse and limits comparability. In a trial in Guatemala, pregnant women and their children up to the age of 7 years were supplemented with a milk-based high protein and energy drink with micronutrients (11·5 gm protein: 163 kcal) or a no-protein, low-energy drink with micronutrients (59 kcal)^(19)^. Children who received the high protein and energy drink had higher cognitive scores at 4–5 years of age, higher scores on tests of numeracy, vocabulary, and reading achievement at 11–18 years of age as well as improved reading and intelligence quotient (IQ) scores in adulthood^(19,36)^. In another study among Indonesian children aged 6–20 months supplemented (for 3 months) with snacks having protein and energy (400 Kcal; 5 g protein/d), a positive effect on motor scores was observed^(37)^. We also observed a significant effect of short-term supplementation with modest amounts of protein on motor scores at 12 months of age. Further, when these Indonesian children were 8–9 years old, the study found a beneficial effect on tests of working memory^(38)^. Rask-Nissilä *et al.* through a sample of 496 Finnish children found that protein intake was associated with improved language scores at the age of 5 years in boys^(18)^.

There are many plausible explanations for why we did not observe significant effects of protein supplementation on neurodevelopment. One of the reasons might be the similarity in the amounts of total protein intake and protein energy ratio between the children in the three study groups. Seemingly adequate protein intake in the control group may be responsible for lack of any additional benefit of moderate or high protein intake. The data from the 24-h dietary recall in a small subsample of children may appear to support this argument. However, there are limitations in terms of extrapolation of the findings of this recall to the entire sample of children studied. We found some positive effect of modest protein supplementation, compared with no-supplement group, on motor scores and infant temperament. This small overall effect might be due to a beneficial effect in subgroups consisting of few participants who would have benefitted from additional protein. Our intervention was focused during second half of infancy, whereas most of the studies that have shown an impact have also covered the period of pregnancy and lactation^(13,19,39)^. The duration of supplementation was also relatively short, that is, 180 d and did not cover the critical period of 24 months entirely. We assessed neurodevelopment at 12 and 24 months of age, whereas most studies that documented an improvement conducted assessments in late childhood. It is well known that brain development tends to be more stable as the age increases, and therefore, any significant effect of an intervention can be reliably detected at later ages^(40,41)^. Another important aspect to consider is that child neurodevelopment is affected by multiple factors, with nutrition being one of them. Therefore, if the intent is to improve neurodevelopment outcome, investment is needed not only in nutrition but also in other aspects of nurturing care, especially responsive care and learning opportunities.

Similar to the present study, a recent systematic review and individual participant data meta-analysis (including data from about 30 000 children from thirteen RCT) found a modest improvement in motor, language and socio-emotional scores in children, aged 6–24 months, receiving small quantity lipid-based supplement^(42)^. The nutritional composition of small quantity lipid-based supplement was similar to the milk–cereal mix provided to infants in the modest-protein group in our study. Further, the review found an enhanced effect of supplementation in populations with high burden of stunted children^(42)^. In the present study, we observed that in the modest-protein group, infants stunted at the time of enrolment had significant improvements in cognitive, motor, language and socio-emotional scores at 12 months of age. Our study also found that supplementation with high protein was not favourably associated with certain aspects of child development. Some studies among high-risk infants observed an association between high-protein supplementation in the very early half of infancy and neurodevelopment impairment at about 24 months of age^(43,44)^. However, it still needs to be tested conclusively whether such an association truly exits and if so, the potential underlying mechanisms need to be identified.

Another possible disadvantage of providing infants with high-protein supplements is the likely increase in the risk of adverse metabolic health, as documented in studies from large cohorts^(45,46)^. A recent review found that children under the age of 2 years from affluent countries often have protein adequacy, and some also have protein consumption in excess of the physiological requirement^(47)^. The authors shared concerns about excess protein and its relation to subsequent development of overweight and obesity^(47)^. It has been suggested that protein energy ratio of 14 % in 12 to 24 months old children should be considered the maximum acceptable level^(48)^. In our study, firstly, the children were from low-resource settings with inadequate access of quality complementary foods and with a high likelihood of gut enteropathy, thereby negatively affecting protein absorption and increasing the demand. Secondly, based on the data on 24-h dietary recall, the protein consumption among the study children did not exceed the 14 % threshold. Nonetheless, we share the concern that one should be cautious while supplementing young children with high-protein diets.

The strength of our study lies in it being a randomised controlled trial done in a community setting. The study achieved high compliance to the supplementation, and the data collection was done by a highly trained team. The neurodevelopment assessments were done in a large sample of infants and children by a team of experienced psychologists. Some limitations included the lack of blinding for the three study groups. However, blinding was ensured for the two supplementation groups by differential coded labelling known only to an independent statistician. At 24 months of age, the behavioural outcomes assessed using CBCL were intended to be presented as proportions, that is, those with internalising and externalising behaviour across the three study groups. However, the numbers of children with clinically significant behavioural problems were very small for a meaningful analysis based on proportions. Another limitation might be in the way we collected data on compliance to milk–cereal mix supplement, that is, by checking empty sachets of the supplement. Empty sachets may not mean that the milk–cereal mix was consumed by the infant enrolled in our study. In the absence of a direct and reliable method of reporting consumption such as directly observed feeding, it may be challenging to ascertain differences in protein intake and utilisation between the groups. We did attempt to minimise sharing by informing the families that the mix was meant only for young children and by providing biscuits/cookies for other children in the household. In about 30 % of the families included in the study, the studied child was of first birth order. This might have reduced the proportion of families in which sharing occurred. However, in spite of these measures, there still remains a possibility of sharing. An additional limitation is that we conducted 24-h dietary recall in a small proportion of infants and children (about 10 % of the total sample) due to limited resources. It may have enhanced our understanding of the findings obtained if a larger subsample of children were assessed for their dietary intakes. Caution should be exercised while drawing interpretations based on data from such a small sample.

### Conclusion

In conclusion, the findings suggest some benefit of short-term supplementation (i.e. 180 d) during infancy with milk–cereal mix containing modest amount of protein and multiple micronutrients on the motor scores and infant temperament soon after supplementation ceased, that is, at 12 months of age, compared with no supplementation. These effects seem to be more pronounced among stunted infants. However, these benefits were not measurable 12 months later, that is, at 24 months of age. The study found no advantage of supplementing infants with milk–cereal mix having high protein, rather a low socio-emotional scores and difficult temperament at 12 months of age was observed. The findings are relevant from the policy perspective as increasing the amount of protein in the supplement increases the cost and has no added advantage. Longer follow-up of infants and children may provide more insights on the effect of nutritional supplementation on neurodevelopment later into childhood and adolescence.

## Supplementary material

For supplementary material accompanying this paper visit https://doi.org/10.1017/S0007114522003944. click here to view supplementary material
