# Supplementary material for: Milk–cereal mix supplementation during infancy and impact on neurodevelopmental outcomes at 12 and 24 months of age: a randomised controlled trial in India
Source: Br J Nutr. 2022 Dec 21;130(5):868–77. doi: 10.1017/S0007114522003944 (PMC10404480; doi:10.1017/S0007114522003944)
Supplement: Supplementary file 1 [file S0007114522003944sup001.doc]

**Supplementary Material**

Milk-cereal mix supplementation during infancy and impact on neurodevelopmental outcomes at 12 and 24 months of age

Ravi Prakash Upadhyay et al.

**Supplementary Figure 1. Proposed pathways through which protein supplementation may influence infant neurodevelopment**

Neural cell proliferation and differentiation

Neurotransmitter production and reuptake

Synaptogenesis

Maintaining electrical efficiency

Protein supplementation

Improved neurodevelopment

Promotion of maturation of neurons, astrocytes and oligodendrocytes

Improved myelination

Enhanced cellular neuroplasticity

Increase Insulin like growth factor 1 (IGF-1)- neurotrophic polypeptide

**Supplementary table 1. Findings of multivariable regression for effect of infant nutritional supplementation on neurodevelopment outcomes at 12 months of age**

|  | **Adjusted mean difference (95% CI)** ^1^ | | |
| --- | --- | --- | --- |
|  | **Modest protein group vs. no supplement group (Ref)**  **(N=762)** | **High protein group vs. no supplement group (Ref) (N=746)** | **High protein group vs. Modest protein group (Ref)** **(N=760)** |
| **BSID composite score** | | | |
| Cognitive | 1.28 (-0.26, 2.83) | 0.91 (-0.64, 2.46) | -0.34 (-1.79, 1.10) |
| Motor | 1.72 (0.49, 2.95) ^2^ | 0.57 (-0.73, 1.87) | -1.14 (-2.39, 0.11) |
| Language | 1.17 (-0.05, 2.39) | 0.26 (-0.99, 1.51) | -0.85 (-2.04, 0.33) |
| Socio-emotional | 0.95 (-0.19, 2.09) | -0.49 (-1.60, 0.62) | -1.48 (-2.52, -0.43) ^2^ |
| **Infant temperament** | | | |
| Total infant temperament score | -2.77 (-4.28, -1.26) ^2^ | -0.74 (-2.31, 0.82) | 2.09 (0.63, 3.56) ^2^ |

^1^ Model adjusted for wealth quintile, maternal age, maternal years of schooling and birth order; BSID- Bayley Scales of Infant and Toddler Development; CI- Confidence interval

^2^ Statistically significant at P<0.01

**Supplementary table 2. Findings of multivariable regression for effect of infant nutritional supplementation on neurodevelopment outcomes at 24 months of age**

|  | **Adjusted mean difference (95% CI)** ^1^ | | |
| --- | --- | --- | --- |
|  | **Modest protein group vs. no supplement group (Ref) (N=810)** | **High protein group vs. no supplement group (Ref) (N=806)** | **High protein group vs. Modest protein group (Ref)** (N=812) |
| **BSID composite score** |  | | |
| Cognitive | 0.23 (-0.74, 1.21) | 0.29 (-0.65, 1.22) | 0.00 (-0.97, 0.97) |
| Motor | 0.31 (-0.81, 1.42) | 0.16 (-0.97, 1.30) | -0.12 (-1.24, 0.99) |
| Language | -1.15 (-2.43, 0.14) | -0.29 (-1.58, 1.01) | 0.75 (-0.55, 2.06) |
| Socio-emotional | -0.41 (-2.73, 1.91) | 0.17 (-2.11, 2.45) | 0.31 (-1.97, 2.59) |
| **Child Behaviour Checklist score** |  |  |  |
| Internalizing behaviour t-score | 0.78 (-0.15, 1.83) | 0.46 (-0.48, 1.41) | -0.43 (-1.41, 0.56) |
| Externalizing behaviour t-score | -0.90 (-2.29, 0.48) | -0.37 (-1.72, 0.98) | 0.57 (-0.82, 1.97) |
| Total problem t-score | 0.19 (-0.91, 1.29) | 0.07 (-0.96, 1.09) | -0.13 (-1.23, 0.98) |

^1^ Model adjusted for wealth quintile, maternal age, maternal years of schooling and birth order; BSID- Bayley Scales of Infant and Toddler Development; CI- Confidence interval

**Supplemental table 3. Data on reported morbidity among infants assessed for neurodevelopment at 12 months of age**

|  | **Intervention** | | **No Supplement Group (N=374)** |
| --- | --- | --- | --- |
|  | **Modest-protein Group (N=388)** | **High-protein Group (N=372)** |
| **Morbidity at 9 mo** |  |  |  |
| Pneumonia^1^ | 4 (1.0) | 2 (0.5) | 5 (1.3) |
| Severe pneumonia^2^ | - | - | - |
| Diarrhea^3^ | 51 (13.1) | 54 (14.5) | 45 (12.0) |
| Fever^4^ | 112 (28.9) | 101 (27.2) | 105 (28.1) |
| Hospitalized since last visit | 1 (0.3) | 1 (0.3) | 1 (0.3) |
| **Morbidity at 12 mo** | | | |
| Pneumonia^1^ | 5 (1.3) | 6 (1.6) | 4 (1.1) |
| Severe pneumonia^2^ | 1 (0.3) | 2 (0.5) | 2 (0.5) |
| Diarrhea^3^ | 4 (1.0) | 5 (1.3) | 3 (0.8) |
| Fever^4^ | 97 (25.0) | 79 (21.2) | 85 (22.7) |
| Hospitalized since last visit | 3 (0.8) | --- | 2 (0.5) |

Values are *n* (%). Data on morbidity collected for the previous 2 weeks from the time of data collection

^1^History of cough (as reported by the mother or caregiver) or difficulty breathing and reported fast breathing or chest indrawing

^2^Pneumonia with general danger signs: not able to breastfeed, feed or drink; lethargy; unconsciousness; or stridor

^3^Diarrhea (as reported by the mother or caregiver) with or without symptoms of dehydration; symptoms of dehydration - not able to drink, lethargy; unconsciousness, restlessness or irritability and sunken eyes

^4^Fever as reported by the mother or caregiver

**Supplemental table 4. Data on reported morbidity among children assessed for neurodevelopment at 24 months of age**

|  | **Intervention** | | **No Supplement Group (N=402)** |
| --- | --- | --- | --- |
|  | **Modest-protein Group (N=408)** | **High-protein Group (N=404)** |
| **Morbidity at 9 mo** |  | | |
| Pneumonia^1^ | 5 (1.2) | 2 (0.5) | 5 (1.2) |
| Severe pneumonia^2^ | - | - | - |
| Diarrhea^3^ | 55 (13.5) | 55 (13.6) | 49 (12.2) |
| Fever^4^ | 100 (24.5) | 109 (27.0) | 98 (24.4) |
| Hospitalized since last visit | 1 (0.3) | 2 (0.5) | 1 (0.3) |
| **Morbidity at 12 mo** |  | | |
| Pneumonia^1^ | 5 (1.2) | 4 (1.0) | 7 (1.7) |
| Severe pneumonia^2^ | 1 (0.3) | - | - |
| Diarrhea^3^ | 44 (10.8) | 57 (14.1) | 54 (13.4) |
| Fever^4^ | 110 (27.0) | 105 (26.0) | 115 (28.6) |
| Hospitalized since last visit | 2 (0.5) | 3 (0.8) | 3 (0.7) |
| **Morbidity at 24 mo** |  |  |  |
| Pneumonia^1^ | 6 (1.5) | 7 (1.7) | 5 (1.2) |
| Severe pneumonia^2^ | 2 (0.5) | 2 (0.5) | 2 (0.5) |
| Diarrhea^3^ | 52 (12.8) | 45 (11.1) | 47 (11.7) |
| Fever^4^ | 107 (26.2) | 90 (22.3) | 95 (23.6) |
| Hospitalized since last visit | 2 (0.5) | 1 (0.3) | 1 (0.3) |

Values are *n* (%). Data on morbidity collected for the last 2 weeks from the time of data collection

^1^History of cough (as reported by the mother or caregiver) or difficulty breathing and reported fast breathing or chest indrawing

^2^Pneumonia with general danger signs: not able to breastfeed, feed or drink; lethargy; unconsciousness; or stridor

^3^Diarrhea (as reported by the mother or caregiver) with or without symptoms of dehydration; symptoms of dehydration - not able to drink, lethargy; unconsciousness, restlessness or irritability and sunken eyes

^4^Fever as reported by the mother or caregiver

**Supplemental table 5. Twenty-four-hour dietary recalls of infants enrolled in the study at 12 and 24 months of age**

|  | **RDA*** | **Modest protein group**  **Mean (SE)** | **High protein group**  **Mean (SE)** | **No supplement group**  **Mean (SE)** |
| --- | --- | --- | --- | --- |
| **At 12 months of age (n=39, 35, 38)** |  |  |  |  |
| Energy, Kcal | 680 | 469 (41.5) | 538.4 (54.7) | 398.4 (48.9) |
| Protein, g ^1^   - Protein-energy ratio (%) | 9.0 | 12.9 (1.2)  11.0% | 17.5 (2.0)  13.0% | 11.7 (1.7)  11.7% |
| Fat, g |  | 11.9 (1.7) | 15.6 (2.6) | 14.6 (2.4) |
| Carbohydrate, g |  | 55.2 (6.4) | 63.9 (6.8) | 53.1 (6.2) |
| **At 24 months of age (n=71, 80, 74)** |  |  |  |  |
| Energy, Kcal | 1110 | 746 (34.4) | 698.6 (28.0) | 765.4 (31.1) |
| Protein, g   - Protein-energy ratio (%) | 12.9 | 20.5 (1.0)  11.0% | 18.9 (0.8)  10.8% | 21.0 (0.9)  11.0% |
| Fat, g |  | 29.2 (1.9) | 28.1 (1.5) | 29.0 (1.6) |
| Carbohydrate, g ^2^ |  | 98.7 (4.1) | 90.2 (3.7) | 102.9 (4.4) |

Statistical significance at P<0.05; SE- standard error; RDA- Recommended Dietary Allowance; *According to the ICMR-NIN Expert group on Nutrient Requirement for Indians, Recommended Dietary Allowances (RDA) and Estimated Average Requirement (EAR)-2020, Available at: https://drive.google.com/file/d/1j3umH5zcJAGNR_WUFwl3-0rBiemYw8DR/view

^1^ Statistically significant difference between high protein and no supplement group; high and modest protein group

^2^ Statistically significant difference between high protein and no supplement group

**Supplemental table 6. Effect of infant nutritional supplementation on BSID composite scores at 12 and 24 months of age in infants stunted at enrolment**

|  | **Modest Protein Group** | **High Protein Group** | **No Supplement Group** | **Adjusted mean difference (95% CI)** ^1^ | | |
| --- | --- | --- | --- | --- | --- | --- |
|  |  |  |  | **Modest protein group vs. no supplement group (Ref)** | **High protein group vs. no supplement group (Ref)** | **High protein group vs. Modest protein group (Ref)** |
| **At 12 months of age (n=89, 76, 78)** | | | | | | |
| Cognitive | 105.2 (9.8) | 101.9 (10.4) | 101.7 (11.9) | 3.33 (0.11, 6.76) ^2^ | 0.05 (-3.58, 3.67) | -3.75 (-6.90, -0.60) ^2^ |
| Motor | 94.5 (8.4) | 91.9 (11.0) | 90.7 (10.7) | 3.94 (0.97, 6.91) ^3^ | 1.35 (-2.22, 4.92) | -2.91 (-5.93, 0.12) |
| Language | 95.8 (8.6) | 92.8 (8.8) | 92.7 (9.8) | 2.82 (0.11, 5.52) ^2^ | 0.12 (-2.75, 2.99) | -3.02 (-5.55, -0.49) ^2^ |
| Socio-emotional | 98.3 (6.6) | 95.8 (8.4) | 96.2 (8.1) | 1.98 (-0.31, 4.27) | -0.61 (-3.30, 2.08) | -2.41 (-4.66, -0.17) ^2^ |
| **At 24 months of age (n=92, 81, 82)** | | | | | | |
| Cognitive | 90.7 (7.1) | 90.4 (7.4) | 89.8 (8.0) | 0.44 (-1.81, 2.68) | 0.79 (-1.57, 3.14) | -0.22 (-2.40, 1.96) |
| Motor | 93.1 (7.6) | 91.9 (8.8) | 91.9 (8.3) | 0.75 (-1.55, 3.04) | 0.01 (-2.63, 2.65) | -1.22 (-3.69, 1.24) |
| Language | 87.7 (9.5) | 88.3 (10.8) | 87.4 (9.6) | -0.10 (-2.93, 2.74) | 0.90 (-2.26, 4.05) | 0.28 (-2.71, 3.28) |
| Socio-emotional | 103.8 (16.2) | 101.0 (14.6) | 102.8 (15.7) | 1.14 (-2.65, 7.10) | -2.21 (-6.86, 2.44) | -2.60 (-7.96, 3.19) |

^1^ Model adjusted for wealth quintile, maternal age, maternal years of schooling and birth order; BSID- Bayley Scales of Infant and Toddler Development; CI- Confidence interval

^2^ Statistically significant at P<0.05

^3^ Statistically significant at P<0.01
